# Quality of Life in Pediatric Patients with Continent Urinary Diversion—A Single Center Experience

**DOI:** 10.3390/ijerph19159628

**Published:** 2022-08-05

**Authors:** Carmen Iulia Ciongradi, Diana Benchia, Cătălina Alexandra Stupu, Codruța Olimpiada Iliescu Halițchi, Ioan Sârbu

**Affiliations:** 1 2nd Department of Surgery—Pediatric Surgery and Orthopedics, “Grigore T. Popa” University of Medicine and Pharmacy, 700115 Iași, Romania; 2 “Sfânta Maria” Emergency Children Hospital, 700309 Iași, Romania; 3 Department of Mother and Child Medicine-Pediatrics, “Grigore T. Popa” University of Medicine and Pharmacy, 700115 Iași, Romania

**Keywords:** continent urinary diversion, Mitrofanoff, qualiveen-30, SF-36 health survey, neurogenic bladder, quality of life

## Abstract

*Background and Objectives*: The advancement of surgical strategies in various types of urological conditions has resulted in improved functional outcomes, but the issues of patient perception and life quality remain difficult to assess, particularly in pediatric populations. We aimed to critically analyze the outcomes of urinary continent diversion in pediatric patients treated in our institution for various bladder conditions. *Materials and Methods*: We conducted a cross-sectional study, reviewing the records of patients treated for bladder evacuation problems between 2003 and 2014, and analyzing the data of those with continent urinary diversion. We used two types of questionnaires to assess the impact on life quality: the Qualiveen-30 and the SF-36 Health Survey. *Results*: The study included one hundred thirty-four patients with bladder conditions, and eight underwent urinary diversion, at a median age of 6.5 years. Seven of them, aged 10–23 years, completed questionnaires, with all seven scoring high on physical functioning scale but low on the social functioning scale. *Conclusions*: Continent urinary diversion remains the treatment of choice in well selected patients, but the results must be considered both in terms of functional outcomes and the impact on these patients’ emotional and mental health.

## 1. Introduction

Nowadays, the evolution of health practices and specific clinical treatments include factors that go beyond physical illness and treatment outcomes, as they attempt to minimize disease consequences in various areas of life and improve social interaction, psychological wellbeing, and overall quality of life (QoL).

While the World Health Organization defines QoL as “an individual’s perception of their position in life in the context of the culture and value systems in which they live and in relation to their goals, expectations, standards and concerns” [1], health-related quality of life (HQoL) refers to the “subjective and objective impact of dysfunction associated with an illness or injury, medical treatment, and health-care policy” [2].

There appear to be differences between the physicians’ assessments and the patients’ perceptions of the impact of the disease and its treatment in many pediatric chronic conditions, so understanding the patients’ experience necessitates tools to measure HQoL.

In children with urinary incontinence, normal mental development in terms of confidence and self-esteem appears to be impaired [3]. There is a subset of such patients who have severe lower urinary tract malformations or congenital or acquired neurologic conditions that make long term urinary continence impossible. Attempts were made over time to treat this chronic disability through extensive surgical procedures, such as the Mitrofanoff technique. The outcome of such medical procedures was measured in terms of physical morbidity or even mortality, but not in terms of patients’ perception of their own QoL.

Paul Mitrofanoff’s description of the “trans-appendicular continent cystostomy”, in 1980, eight years after Lapides described the clean intermittent catheterization technique (CIC), revolutionized the treatment of neurogenic bladder [4,5]. The Mitrofanoff principle proposed a novel idea in which the bladder could be emptied via a route other than the urethra. This was especially critical when the urethra couldn’t be used or the patient needed CIC for the rest of his life. The appendix was the only segment of bowel used at first, and the bladder neck was usually closed. Because the appendix is not always available, various techniques based on the Mitrofanoff principle have been described with other intestinal segments over time: the transverse ileal tube (Yang-Monti technique), the double tube (Monti technique), and the Casale technique (Monti spiral technique) [6].

Despite its origins as a technique for restoring continent bladder emptying in children with congenital neuropathic bladder dysfunction, the Mitrofanoff channel is now used for a variety of purposes. These include: complex urethral strictures due to location, non-viable urethral reconstructive surgery, traumatic loss with failed reconstruction, congenitally absent urethra, posterior urethral valves, Prune Belly syndrome, bladder or cloacal exstrophy-epispadias complex, and idiopathic dysfunctional bladder [7,8,9,10].

With these definitions in mind and knowing that the use of CIC on a daily basis causes significant changes in the lives of these patients, affecting their social routines, professional activities, and other aspects of their lives, the goal of this work is to identify the urological pathologies for which Mitrofanoff surgery is indicated, and to analyze the emotional impact on HQoL in patients who underwent this procedure, and to compare the results with data obtained from other studies in the literature.

Our hypothesis was that, despite successful surgeries, the QoL of patients with urinary diversion could be impaired not only by the disease, but also by the procedure itself.

## 2. Materials and Methods

We conducted a cross-sectional study of all patients with neurogenic bladder associated with myelomeningocele, neurogenic-like bladder due to other etiology, bladder exstrophy, posterior urethral valves, and post-traumatic urethral stenosis who were treated in the Pediatric Surgery Department of the “Sfânta Maria” Children’s Emergency Hospital Iași, between January 2003 and December 2014. Patients with a continent urinary diversion (CUD) based on the Mitrofanoff principle were selected for the study, and data from their medical records was retrospectively evaluated.

Patients with urinary diversion in use, and who had undergone surgery during childhood, met the inclusion criteria. Exclusion criteria encompassed cognitively impaired patients unable to answer the questions.

Legal guardians provided informed consent, and the Emergency Children’s Hospital Committee of Ethics authorized the research.

To assess the impact on HQoL in patients who underwent this procedure, we used two types of questionnaires: the Qualiveen-30 questionnaire, and the SF-36 Health Survey. The two surveys were sent through the mail.

The Qualiveen-30 questionnaire is a specialized instrument for assessing the HQoL of patients with neurological urinary diseases; it was originally designed in French, validated and first administered to patients with spinal cord injuries and multiple sclerosis. The questionnaire consists of 30 questions measuring the impact of urinary difficulties on QoL across four dimensions:

- impediments and constraints (9 questions)
- limitations (8 questions)
- concerns (8 questions)
- emotional impact (5 questions) [11,12].

Each Qualiveen test result is calculated by averaging sum of each question group’s points. Each group of questions from the four aforementioned categories contributes equally to the final score. Answers are rated as a 5-point Likert scale, ranging from 0 (no impact of urinary problems on HQoL) to 4 (major impact). Each questions group will give a score, and the final test score will be the average of the four areas.

Scores range from 0 to 4, and are interpreted as follows:

0—No effect on quality of life;
1—Slight impact on quality of life;
2—Moderate impact on quality of life;
3—Significant impact on quality of life;
4—Significant impact on quality of life.

The SF-36 Health Survey, an abbreviated version of the Medical Outcome Study (MOS) questionnaire, contains 36 questions and was developed as an indicator of health status in the general population, useful for monitoring the condition of patients with single or multiple pathological conditions and comparing their status to that of the general population. The SF-36 assesses eight concepts of quality of life: physical functioning, which assesses an individual’s perception of their ability to perform physical tasks; role physical, which examines an individual’s perception of their physical limitations; bodily pain, a domain that examines a subject’s experience of pain and how this impacts their quality of life, general health and vitality, which examines a subject’s perception of their health and the impact of their constitution on their life; social functioning, which investigates the impact of health on a subject’s ability to interact normally with peers, family, and friends; as well as emotional role and mental health, which provide assessments of how a person’s health influences their mood [13].

The questions pertinent to each section are first identified. The scores are then computed based on the numeric values and keys that have been specified. Each question is graded on a scale from 0 to 100. Each field’s score is computed by dividing the number of points earned by the number of questions in that field, with a high score indicating a better state of general health.

The values obtained for each question in a group that addresses one specific aspect of the health status are then averaged together to yield a final score for each of the eight issues assessed. A score of 100 represents the best possible level of functioning, so the lower the obtained value, the more affected that domain will be (Table 1). 

## 3. Results

### 3.1. Patient and Urological Condition Characteristics

**Figure 1:**
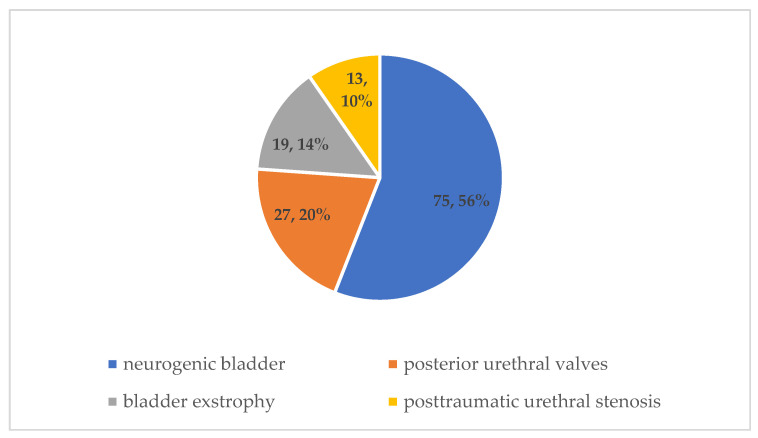
Distribution of pathologies that may require a continent urinary diversion.

The initial selection of 134 patients for this study included 75 individuals with neurogenic bladder,19 with bladder exstrophy, 27 with posterior urethral valves, and 13 with post-traumatic urethral stenosis (Figure 1). 

A Mitrofanoff procedure was performed on only eight patients out of a total of one hundred thirty-four, including two cases of neurogenic bladder secondary to myelomeningocele, one case of neurogenic-like bladder, two cases of bladder exstrophy, two cases of posterior urethral valves, and one case of post-traumatic urethral stenosis.

Except for the eldest participant, who had then completed high school, all of the patients in the study were enrolled in school. There were five males and three females receiving a CUD according to the Mitroffanof principle. A summary is presented in Table 2.

### 3.2. QoL Outcomes 

We were able to contact seven patients out of a total of eight, all aged 10–23 years, and we asked them to complete two types of questionnaires: the Qualiveen-30 and the SF-36 Health Survey.

The Qualiveen-30 questionnaire asks patients about four aspects of their lives: obstacles and constraints (questions 1–9); limitations (questions 10–17); concerns (questions 18–25); and emotional impact (questions 26–30). The score for each area is calculated as an average of the answers to the corresponding questions; a lower score indicates a good quality of life (i.e., no limitations, constraints, fears, or emotional impact) and a higher score indicates a poor quality of life.

**Figure 2:**
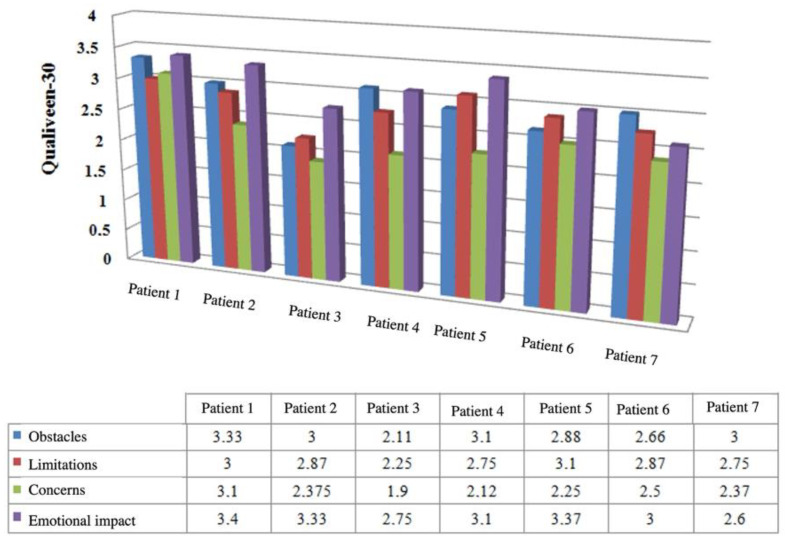
Qualiveen questionnaire results.

Obstacles in daily activities affect patients’ QoL postoperatively, with four of the seven patients scoring higher than 3 in this area (Figure 2). The restrictions imposed by performing sterile intermittent catheterizations alter patients’ lifestyles; in this area, there are two scores >3. Only one patient scored above 3 in terms of concerns, with the remaining patients having reasonable values. The emotional impact section yielded the best results, with five of the seven patients scoring 3 or above, and the other two scoring very close to 3.

The SF-36 Health Survey evaluates general health in eight categories: physical function, role physical, physical pain, general state of health, vitality, social functioning, emotional role, and mental health. This type of questionnaire is divided into two steps for the purposes of analysis. The questions pertinent to each section are first identified. The scores are then computed based on the numeric values and keys that have been specified. Each question is graded from 0 to 100. Each field’s score is computed by dividing the number of points earned by the number of questions in that field, with a high value of the score signifying a better state of general health.

**Figure 3:**
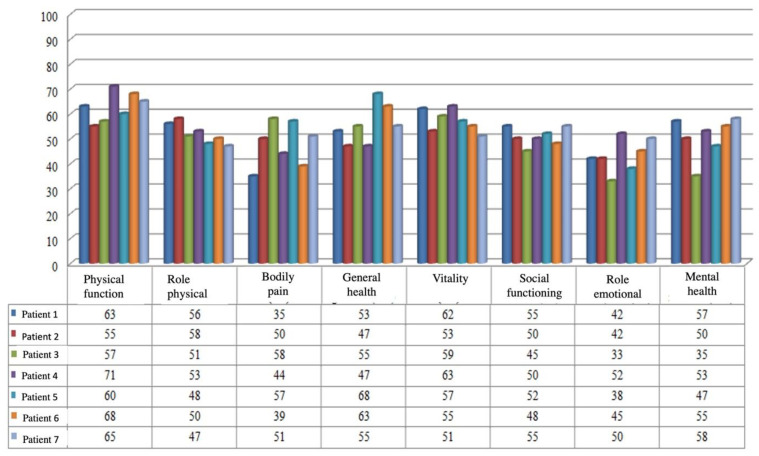
Results of SF-36 questionnaire.

Physical functioning was good in all seven patients, indicating a reduced constraint in daily activities (Figure 3); patients stated that they were not limited to climbing a floor or carrying groceries, but that as the complexity of the activity increased, they became more limited. The role physical represents the second scale, which sums up activity limitations caused by both physical and emotional impairment. Emotional functioning influenced the limitation of daily activities because it had lower values than did the physical functioning scale. The emotional role scale had the lowest values, indicating that some emotional issues, such as depression or anxiety, as reported by patients, had a negative impact on overall health.

Furthermore, patients’ physical pain interferes with their daily activities, but it appears to have a smaller impact on their overall health than does the emotional component, as the character and duration of the physical pain change over time.

The vitality scale demonstrates that patients’ vitality and energy are affected by both physical and emotional pain.

Physical health and/or emotional problems have significantly impacted daily activities with family, friends, and neighbors, according to low values on the social functioning scale.

Patients’ mental health may suffer as a result of their reliance on continent urinary diversion and the emergence of new limitations upon their lives.

The general state of health is an assessment of their own health, with the majority believing that their health is neither very good nor good, but satisfactory, and that their health deteriorates more frequently than does others’.

## 4. Discussion

Although the primary goals of urological management of patients with dysfunctional voiding are to preserve renal function and prevent complications, urinary continence is an important aspect that has a significant impact on the patient’s QoL and long-term independence [14,15,16]. Despite this, few studies on the impact of surgical treatment on these patients’ QoL have been conducted, and they have returned contradictory results [17,18,19]. Different populations may experience different aspects of QoL, because the latter includes social, psychological, and physical aspects. Furthermore, measuring quality of life in children and adolescents can be difficult.

In this study, we used two validated scales to assess a special group of patients receiving a CUD—pediatric patients. The Qualiveen 30 questionnaire revealed that the patients’ main issues are their reliance on CUD and difficulties performing an emptying procedure outside of their own environment. According to the responses, the main reasons for reduced quality of life are reliance on continent urine diversion and personal hygiene issues outside home, which appear to bother them even more than do occasional urine leaks. Furthermore, the difficulties associated with performing sterile intermittent catheterizations alter patients’ lifestyles. Concern and emotional impacts primarily affect patients’ QoL, with the fear of aggravating their bladder problems lurking in the background. Concerns and limitations are areas with low scores; therefore Mitrofanoff’s intervention treated the patient’s urological issues, incontinence, and the occurrence of frequent episodes of urinary tract infections, fears of which did not interfere too much with daily activities. The high results in the categories of limitations and emotional impact indicate that the constraints are due to dependence on continent urinary diversion rather than medical factors.

The SF-36 Health Survey results revealed that the CUD had no significant impact on physical functioning, with patients able to perform their usual activities. More complex actions reveal an impact and, to some extent, limit them. The CUD appears to have the greatest impact on our patients’ emotional functioning, with the scale of this aspect having the lowest values, and with feelings of anxiety or depression negatively impacting their overall health. This effect is more significant than physical pain, even though some patients experience both. Both emotional and physical pain have an impact on the patients’ energy and vitality in varying degrees, making it difficult to distinguish between the two.

Smith et al. assessed 19 patients over the age of 16 using the SF-36 Health Survey, version 2. Physical Functioning (PF = 50.4), Role Physical (RP = 53.8), Bodily Pain (BP = 55.6), Vitality (VT = 56.9), Social Functioning (SF = 51.5), Role Emotional (RE = 52.2), and Mental Health (MH = 54.6) all scored higher than in the normal population (normal = 50.0), and the same results were found when compared to age-matched controls [20].

Furmincelli et al. reviewed 13 studies that reported on QoL in patients reliant on Mitrofanoff catheterization, which had been reported to be good in the majority of studies reviewed [21,22].

Lima et al. evaluated QoL in patients with neurogenic bladder who had urological reconstructive surgery. They assessed patient reported outcomes using the SF-36 Health Survey and the Qualiveen questionnaire and discovered statistically significant improvements in all domains [22].

According to a recent study on 25 cases with CUD conducted by Chavarriaga et al., the procedure is associated with good health quality of life, global satisfaction, ease and painless catheterization, adequate self-perception of cosmetic outcomes, and a low complication rate, indicating that it is a safe and viable option [23].

Another factor that may have an impact on QoL is the procedure’s complication rate. Despite all of the modifications, CUD using the Mitrofanoff principle remains a complex procedure with a high complication rate [24]. Despite all efforts to improve the technique, stomal stenosis remains the most commonly reported complication in the literature, with a stenosis rate ranging from 6 to 39 percent [24]. However, in many cases, channel dilation or simple endoscopic procedures can be used to manage this conservatively [25], with complex revisions being sometimes required regardless of the conduct type (appendix, bowel, bladder flap), also taking into account that this type of surgery is aimed at growing bodies with changing pattern of body habits [26,27]. The findings are consistent with our research, which discovered a relatively high rate of complications such as stenosis, recurrent urinary tract infection, incontinence, and bladder stones.

As with other chronic and debilitating conditions, assessing QoL should always be part of assessing symptoms and monitoring treatment outcomes, because the short-term decision of such a surgery comes with long-term consequences for the patient’s life, emerging as a critical component [28]. HQoL measurement provides a multidimensional view of the patient’s life, including physical, mental, emotional, and social well-being.

The achievement of social continence is a significant milestone in the lives of these patients. Continence is an expected social behavior [29]. It has been postulated that incontinence has a negative impact on children’s development of confidence and self-esteem [3]. However, the impact of their condition on their daily lives does not end with the operation. A surgeon should be aware that, while the intervention was successful in making patients ‘dry,’ committing to intermittent catheterization for the rest of one’s life may potentially be replacing one problem with another [30]. These impacts also frequently involve family members or caregivers, who usually accompany the patient during treatment and are in charge of the procedure’s execution.

These findings are similar to ours. Our patients’ perceptions of their health appear to be generally positive, with a few exceptions. Even if the intervention significantly improved their physical functioning and reduced the limitations caused by their urological condition, the emotional impact of the continent urinary diversion appears to have a significant impact on their QoL and social functioning. It should be noted, however, that this instrument assesses QoL from the patient’s point of view.

The procedure’s disadvantages are outweighed by the procedure’s excellent continence rates, together with improved QoL and cosmesis for many patients. Establishing a CUD has a major emotional consequence. It would be very advantageous to provide psychological counseling to these individuals in order to support their mental health and emotional well-being.

The limitations of our study include its retrospective nature, as well as its small sample size. A prospective study comparing QoL before and after CUD surgery would help to answer many of the remaining questions about the procedure.

## 5. Conclusions

Patients with dysfunctional voiding frequently require mandatory CIC, and sometimes, surgery to create a CUD is required. The Mitrofanoff principle remains a viable treatment option in selected cases of various urologic or neurologic conditions affecting bladder emptying.

Even though the primary goals of urological management of patients with bladder dysfunction are to preserve renal function and to prevent complications, the patient’s evolution in terms of continence, as well as the family’s compliance with post-operative care and the child’s integration into the community, are all factors that must be considered in the management of these patients. 

## Figures and Tables

**Table 1:** Information About SF-36 Health Status Scales and the Interpretation of Low and High Scores [13,14].

			Meaning of Scores	
Concepts	No. of Items	No. of Levels	Low	High
Physical functioning	10	21	Limited a lot in performing all physical activities including bathing or dressing	Performs all types of physical activities, including the most vigorous, without limitations due to health
Role limitations due to physical problems	4	5	Problems with work or other daily activities as a result of physical health	No problems with work or other daily activities as a result of physical health in the past 4 weeks
Social Functioning	2	9	Extreme and frequent interference with normal social activities due to physical and emotional problems	Performs normal social activities without interference due to physical or emotional problems over the past 4 weeks
Bodily pain	2	11	Very severe and extremely limiting pain	No pain or limitations due to pain over the past 4 weeks
General mental health	5	26	Feelings of nervousness and depression all of the time	Feels peaceful, happy, and calm all of the time over the past 4 weeks
Role limitations due to emotional problems	3	4	Problems with work or other daily activities as a result of emotional problems	No problems with work or other daily activities as a result of emotional problems over the past 4 weeks
Vitality	4	21	Feels tired and worn out all of the time	Feels full of pep and energy all of the time over the past 4 weeks
General health perceptions	5	21	Believes personal health is poor and likely to get worse	Believes personal health is excellent

**Table 2:** Patients included in our study.

Age	
Age when CUD was performed	Median 6.5 (4–13)
Age at the moment of the study	Median 13.5 (8–23)
Male/Female ratio	1.6:1
Primary diagnosis and indication for CUD	
Neurogenic bladder	2
Neurogenic-like bladder	1
Bladder exstrophy	2
Posterior urethral valves	2
Post-traumatic urethral stenosis	1
Complications	
stenosis	5
Urinary tract infection	4
Incontinence	1
Bladder stones	2

## Data Availability

Data is contained within the article.
